# Hormonal Modulation in Aging Patients with Erectile Dysfunction and Metabolic Syndrome

**DOI:** 10.1155/2013/107869

**Published:** 2013-12-28

**Authors:** Inês Campos Costa, Hugo Nogueira Carvalho, Luís Pacheco-Figueiredo, Inês Tomada, Nuno Tomada

**Affiliations:** ^1^Faculty of Medicine, Universidade do Porto, Alameda Professor Hernâni Monteiro, 4200-319 Porto, Portugal; ^2^Department of Urology, São João Central Hospital, Alameda Professor Hernâni Monteiro, 4200-319 Porto, Portugal; ^3^School of Health Sciences, Life and Health Sciences Research Institute (ICVS), University of Minho, Campus de Gualtar, 4710-057 Braga, Portugal; ^4^Department of Experimental Biology, Faculty of Medicine, Universidade do Porto, Alameda Professor Hernâni Monteiro, 4200-319 Porto, Portugal; ^5^Institute for Molecular Cell Biology, Universidade do Porto (IBMC), Rua do Campo Alegre 823, 4150-180 Porto, Portugal; ^6^Faculty of Biotechnology, Catholic University of Porto, Rua Dr. António Bernardino de Almeida 400, 4200-072 Porto, Portugal

## Abstract

Erectile dysfunction (ED), metabolic syndrome (MetS), and hypogonadism are closely related, often coexisting in the aging male. Obesity was shown to raise the risk of ED and hypogonadism, as well as other endocrinological disturbances with impact on erectile function. We selected 179 patients referred for ED to our andrology unit, aiming to evaluate gonadotropins and estradiol interplay in context of ED, MetS, and hypogonadism. Patients were stratified into groups in accordance with the presence (or not) of MetS and/or hypogonadism. Noticeable differences in total testosterone (TT) and free testosterone (FT) levels were found between patients with and without MetS. Men with MetS evidenced lower TT circulating levels with an increasing number of MetS parameters, for which hypertriglyceridemia and waist circumference strongly contributed. Regarding the hypothalamic-pituitary-gonadal axis, patients with hypogonadism did not exhibit raised LH levels. Interestingly, among those with higher LH levels, estradiol values were also increased. Possible explanations for this unexpected profile of estradiol may be the age-related adiposity, other estrogen-raising pathways, or even unknown mechanisms. Estradiol is possibly a molecule with further interactions beyond the currently described. Our results further enlighten this still unclear multidisciplinary and complex subject, raising new investigational opportunities.

## 1. Introduction

Erectile dysfunction (ED) is the persistent inability to attain and/or maintain an erection of sufficient rigidity for sexual intercourse. Its prevalence has a tendency to increase over time [1], being expected a worldwide increase of 12.0% from 1995 to 2025 [2]. An observational study revealed that 12.9% of Portuguese men suffer from ED, with 5.8% describing it as moderate/severe, and that ED was most prevalent amongst men over the age of 60 [3]. ED has been linked to metabolic syndrome (MetS) [4], which is an assembly of cardiovascular (CV) and metabolic risk factors, such as visceral adiposity, insulin resistance/diabetes, high blood pressure, and dyslipidemia. Although several definitions of MetS have been devised, the one proposed by the National Cholesterol Education Program-Adult Treatment Panel III [5] emerged as the most widely used definition, due to its simplicity and superiority as a predictor of secondary outcomes. Its prevalence in Portuguese male population is 18.7% [6], which is superior to other European countries [7].

Aging and age-related comorbidities are strongly associated with the increased prevalence of ED [1, 8], MetS [6], and hypogonadism [7]. All these conditions often coexist in the same patient [9, 10], whilst hypogonadism and ED have been demonstrated to increase the risk of MetS [9, 11–15], supporting the idea of a multifactorial/directional endocrinological imbalance that occurs in specific subsets of ED patients. Elderly men are prone to develop late onset hypogonadism (LOH), a condition characterized by a progressive testicular impairment associated with specific sexual symptoms (amongst them, ED) and a deficiency of serum T levels [16, 17]. Several studies support LOH, unveiling that T levels decrease along aging [14, 16, 18, 19].

Obesity by itself contributes to a wide range of endocrine disturbances, such as reduced T levels. Indeed, among ED patients, obese men present lower T levels compared to those observed in the elder [15]. One of the most plausible mechanisms by which obesity contributes to T levels decline is the adipose tissue dependent aromatization of T to estradiol [15]. In line with these remarks, hormonal ED etiology might not be confined to androgen deficiency, as isolated high estradiol levels have been reported in some ED patients [8, 20].

In addition to sexual hormones disturbances, several other endocrinological imbalances might be found in ED patients. ED has been related with glandular anomalies, such as hyper- and hypothyroidism [21, 22]. However, the relationship between the latter and ED is still controversial [21–23]. Nonetheless, it is unlikely that ED associated with hyperthyroidism is owed to hypogonadism, as no thyroid hormone-dependent alteration in calculated free testosterone (FT) levels has been described [21]. Severe hyperprolactinemia has been also reported as a cause of ED, since it may compromise sexual desire and inhibit T secretion [22, 24], an outcome that is potentially reversible by treating the underlying disorder [24].

Hypogonadism and MetS strongly increase the risk of ED at any age and both are risk factors for CV disease [9, 25–27]. From several studies in this area, the importance of understanding the complex interactions between those entities became clear, as well as the underlying modulating factors. In this study, we describe the hormonal milieu of a Portuguese population with ED, comparing MetS to non-MetS groups and correlating T levels with the number of MetS parameters. Moreover, we analyze the hypothalamic-pituitary-gonadal (HPG) axis in ED patients with and without hypogonadism, aiming to describe and evaluate nonexpected hormonal alterations in this axis and further uncover the relation between these three increasingly prevalent conditions.

## 2. Materials and Methods

We selected 179 Caucasian patients referred to our andrology unit for ED between January 2008 and March 2012. All of them gave their written informed consent and had a complete hormone assessment available. A known history of neurological disease, pelvic trauma, major psychiatric disorder, hepatic failure, end-stage renal disease, or drug abuse was an exclusion criterion. A standardized health questionnaire covering medical anamnesis (including sexual history), CV risk factors, smoking and alcohol intake history and current medication was obtained. All patients underwent a standardized physical examination protocol. Anthropometric evaluation, including weight, height, and waist circumference (WC), was acquired by the same technician with the subjects in light clothing and barefoot. Blood pressure was measured in the right arm using an automatic manometer (DINAMAP Procare 300, GE, UK) in the sitting position after a 10-minute rest period. Blood analysis was performed using samples of venous blood collected between 8:00 and 10:00 a.m., after a 12-hour overnight fasting period.

The following measurements were made by routine laboratory methods: triglycerides (Trig), high-density lipoprotein cholesterol (HDL), low-density lipoprotein cholesterol (LDL), total cholesterol, serum glucose, and albumin. Total testosterone (TT), sex hormone-binding globulin (SHBG), estradiol, prolactin, follicle-stimulating hormone (FSH), luteinizing hormone (LH), and insulin were determined by chemiluminescence with a commercially available kit (Cobas; Roche Diagnosis GmbH, Manheim, Germany). Triiodothyronine (T3), thyroxine (T4), and thyroid-stimulating hormone (TSH) were determined by chemiluminescence with a commercially available kit (Abbott Diagnostics Division, Princeton, NJ, USA). Free testosterone was calculated using the *Free and Bioavailable Testosterone calculator*, developed at the Hormonology Department, University Hospital of Ghent, Belgium (http://www.issam.ch/freetesto.htm). The electronic process of each patient and available digitalized records were also consulted. Body mass index (BMI) was calculated based on weight in kilograms and height in meters (kg/m^2^). Participants were considered obese if BMI was ≥30 kg/m^2^. The presence of three or more of the following criteria defined MetS, according to NCEP-ATPIII (2002): central obesity (WC >102 cm), hypertriglyceridemia (Trig ≥150 mg/dL or treatment), low HDL cholesterol (<40 mg/dL or treatment), hypertension (HT, blood pressure ≥130/85 mmHg or treatment), and fasting serum glucose ≥110 mg/dL [5]. Hypogonadism was defined as TT below 3.50 ng/mL (11.10 nmol/L) or calculated FT below 0.072 ng/mL [28]. We considered normal LH levels between the normal range of 1.70 to 8.60 mUI/mL. Hypothyroidism was defined as TSH levels >4.94 *μ*U/mL and T4 levels in the normal range or <0.70 ng/dL. Hyperthyroidism was defined as TSH levels <0.35 *μ*U/mL, T3 levels >3.71 pg/mL, and T4 levels >1.48 ng/dL. Hypo- and hyperinsulinemia were considered at insulin levels inferior to 2.60 *μ*U/mL and superior to 24.90 *μ*U/mL, respectively.

### 2.1. Statistical Analysis

Absolute and relative frequencies were used to describe categorical variables as well as median and percentiles 25 and 75 were applied in the description of continuous variables.

Chi-square and Kruskal-Wallis tests were used to evaluate the differences between the subgroups with and without MetS, respectively, for categorical and continuous variables. We considered the differences statisticaly significant when *P* < 0.050.

All data analyses were performed using STATA software, version 9.2.

## 3. Results

From the 179 patients who were engaged in this study, 28 subjects were excluded due to insufficient data. The remaining 151 subjects had a median age, BMI, and WC, respectively, 56 years (P25–P75: 50–62), 27.9 kg/m^2^ (25.2–30.5), and 104.0 cm (97.0–111.0). Current or past alcohol consumption was absent in the majority of patients (61.9%), having only 36.8% reported a current frequent intake (until 37 gr of alcohol per day) and 1.8% history of past intake. Smoking habits were majorly absent (36.7%) or in the past (42.0%), having only 21.3% reported being active smokers. Further population characteristics are contemplated in Table 1.

Concerning the hormonal profile, the median TT, FT, SHBG, LH, and estradiol levels were 4.5 ng/mL (P25–P75: 3.5–5.6), 0.102 ng/mL (0.074–0.156), 35.8 nmol/L (27.3–48.6), 4.0 ng/mL (2.7–5.9), and 28.5 ng/mL (22.0–37.8), respectively. Levels of FSH, TSH, T3, and T4 were also measured and are described in Table 1. Hypothyroidism was not present in any of the patients, whereas hyperthyroidism was present in one. Hyperprolactinemia was present in 15 patients (8.2%) and hyperinsulinemia in 5 (7.4%).

MetS, according to NCEP-ATPIII criteria, was present in 49% of our patients. These patients evidenced significantly higher calculated FT levels of 0.1225 ng/mL (0.0738–0.6530), when compared to patients without MetS (*P* < 0.001). Conversely, patients without MetS evidenced median TT of 5.1 ng/mL (4.1–5.9) and median SHBG of 40.2 nmol/L (31.4–55.0), which were significantly higher in comparison to those with MetS (*P* = 0.001 and *P* = 0.003, resp.). Comparison measurements are further described in Table 1.

Age, TSH, FSH, and estradiol levels presented no significant differences between patients with or without MetS (*P* = 0.790, *P* = 0.634, *P* = 0.164, *P* = 0.164, resp.). No differences were observed in median LH levels between patients with or without MetS (*P* = 0.062).

**Figure 1:**
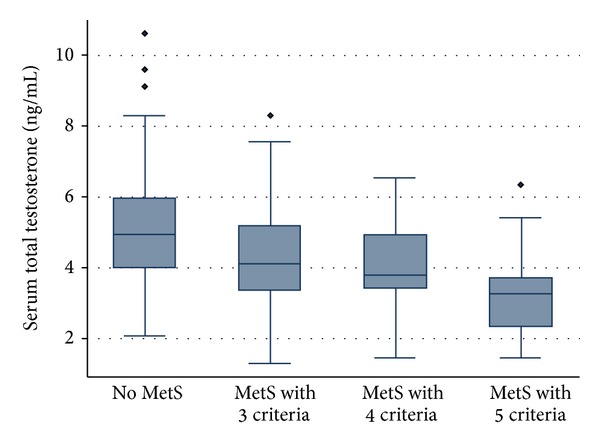
Box-plot representation showing a statistical significant decrease of the total testosterone levels (vertical axis) with the increase of the metabolic syndrome (MetS) parameters (horizontal axis) (*P* < 0.001).

The relationship between hypogonadism and MetS was also evaluated. Hypogonadism was present in 23.2% of the patients, and no differences in its frequency were found between patients with and without MetS (*P* = 0.953). However, TT levels were decreased consistently with the increase of the number of parameters of MetS (*P* < 0.001) (Figure 1). Moreover, multivariate regression analysis demonstrated that within MetS, WC (*P* = 0.017) and hypertriglyceridemia (*P* = 0.050) were independently associated with a decrease in serum T levels. In spite of such observations, the presence of MetS did seem not to influence TT and FT levels amongst patients with hypogonadism. Multivariate regression analysis also demonstrated that the MetS is a determinant independent of lower TT levels independent of the other model variables such as age, alcohol consumption, and smoking habits (beta coefficient: −0.70; 95% confidence interval: −1.29, −0.10).

The relationship between MetS with LH levels was evaluated and is presented in Table 2. LH levels were within normal range or decreased in 136 patients, regardless of the presence of MetS. Moreover, amongst patients with hypogonadism, LH levels did not vary with the MetS presence (*P* = 0.844).

**Figure 2:**
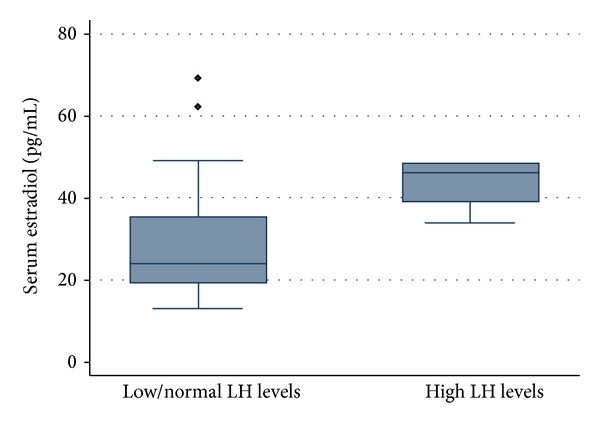
Box-plot representation showing a statistical significant increase of the estradiol levels (vertical axis) with the increase of the luteinizing hormone levels (LH) (horizontal axis) (*P* = 0.033).

On the other hand, as shown in Figure 2, estradiol levels varied in patients with hypogonadism, which was related to dissimilarities in LH levels (*P* = 0.033). In these patients with hypogonadism, we characterized the estrogen levels according to the axis response (low or normal LH levels versus raised LH levels). Indeed, while estradiol levels increased, LH levels were raised (normally functioning axis) and dropped while the LH levels were normal or low.

## 4. Discussion

Hypogonadism and MetS are strongly associated [12, 13, 16], having even been demonstrated that with the increasing number of MetS parameters there is a proportional raise in the incidence of hypogonadism [29]. Although hypogonadism was present in 23.2% of our patients, no noticeable differences were observed among those with or without MetS.

In the recent years, several studies unveiled that the increasing number of MetS components is inversely associated with T levels [12, 13, 27, 30]. Accordingly, our population with hypogonadism and MetS showed a decrease in T levels with the increase in the number of MetS parameters. Thus, although the presence of MetS did not prove to be a significant determinant of hypogonadism, as it did not lead to a decline in T levels, in MetS patients with already established hypogonadism, the increasing number of MetS features was associated with further decline in T.

In the setting of MetS, hypertriglyceridemia and increased WC have been reported as the most important determinants of hypogonadism [10, 16]. Accordingly, in patients with MetS, these two features revealed the major influence on decreasing T levels, with the former being the strongest independent conditioning factor. In fact, recent literature consistently associates obesity not only with higher risk of hypogonadism [4, 6, 27] but also with lower T levels [8]. Visceral adiposity has been particularly related with reduction of T and SHBG levels (independent of other metabolic disorders) [15, 27]. In this study, WC was one of the MetS parameters with the greatest influence in T levels decrease, presenting itself as a strong risk factor for hypogonadism development. Furthermore, the metabolic and hormonal profile of our population is not only consistent with ED presence but also reinforces the usefulness of MetS screening for ED and CV disease prevention [9, 25–27].

Aiming to establish further relations amongst the conditions described above, several authors observed that the MetS-related T decline was not accompanied by an increase in pituitary LH levels, suggesting impairment in gonadotropin secretion [27]. The term *mixed hypoandrogenism* (primary and secondary) was proposed for this phenomenon [9, 31]. The molecules behind this smoothing compensatory effect of GnRH/LH are still unknown, but estrogens and insulin, as well as leptin, TNF-*α*, and other adipokines, were proposed candidates [15, 27]. An alternative etiopathogenic explanation for this phenomenon proposes that fat stores undertake an increase aromatization of androgens, therefore raising estrogen levels [9, 15], which in turn decrease LH secretion [9]. Hence, aging being associated with an increase in adipose tissue accretion, a link between age and hypogonadism was established [32]. However, in opposition to *mixed *hypoandrogenism definition, our MetS patients did not evidence changes in the median LH levels compared to non-MetS ones. This was also true when only the patients with hypogonadism were considered, suggesting that other factors than MetS and obesity, such as age or any of the molecules described above, might have a stronger influence on the HPG axis. It is likely that the underlying ED, a common feature of all of our patients, might play a multi-factorial masking role. Although possible unforeseen confounders might also be influencing this relation, the greatest efforts were made in order to exclude patients with known underlying pathologies or comorbidities that could influence the hormonal results.

Theoretically, our data contradicts the concept that estradiol exerts a negative feedback on hypothalamic GnRH secretion [28], as it would not be expected to find raised estradiol levels concomitant with raised LH levels. We hypothesize that, in our group of patients with MetS that did not present estrogenic HPG axis-attenuation effect, obesity is playing an important role in the estradiol levels increase. Nevertheless, we have just a few patients without an estrogenic HPG axis-attenuation effect and, amongst them, there were not enough MetS cases to accurately test this hypothesis in a MetS setting. On the other hand, when considering data from patients with an estrogenic HPG axis-attenuation effect but without MetS, possible explanations may imply additional molecules, age-related fat deposits, or other estrogen-raising mechanisms. However, even in this setting, the absence of estradiol effect on LH secretion is still puzzling. Thus, taking into account that high estradiol levels have already been described as the only abnormality in a subset of patients with ED, the hypothesis that the later might not only be caused by androgen deficiency is becoming increasingly evident [8, 20]. Furthermore, it has been reported that the chronic exposure to phosphodiesterase type 5 inhibitors (PDE5i), widely used for the treatment of ED, may influence serum estradiol levels [33, 34]. Even though we did not evaluate the influence of PDE5i on the hormonal axis, we cannot exclude the role these drugs might have in the serum T : estradiol ratio of our patients.

When expanding this analysis for a broader endocrinological spectrum, one must consider that even though a very limited number of cases of dysthyroidism were found in our population, thyroid disorders (specially hyperthyroidism) have been related to ED and hypogonadism, and so must be considered in a sexual-dysfunction setting [21, 22]. It is clear from the current literature that collecting a more thorough hormonal panel might be a wise approach to further uncover hormonal relations.

Our study has several limitations, one being its retrospective design. It is possible that some confounders have not been traced, therefore influencing the results. Nevertheless, a thorough analysis of the patients was made in order to exclude underlying pathologies or comorbidities that could alter the results. Moreover, the sample size was sometimes a limiting factor to test some hypothesis.

We concluded that in ED patients with hypogonadism and MetS, the attenuated response of HPG axis (normal or low LH levels) might not always be due to an underlying adiposity-dependent estrogen-raising effect. The lower estradiol levels observed in this peculiar group of patients suggest that they may be influenced by mechanisms that are not yet unveiled. Similarly, the increased estradiol levels in patients with hypogonadism and a normally responsive HPG axis (raised LH levels), mainly when MetS is not present, remain to be elucidated. In addition, the basis for estradiol negative feedback mechanism on the hypothalamic GnRH secretion and consequently on the HPG axis should be further investigated to clarify the concomitant increase of LH and estradiol. In the meantime, our findings indicate that ED, aging, and estradiol might have a stronger connection than what is currently described in the literature.

Overall, this study underlines the importance of the collection of a full hormonal panel in ED men, as well as a detailed clinical history, to exclude the presence of other hormonal disturbances than hypogonadism and MetS. Our results yield further insights into this still unclear multidisciplinary and complex subject and raise new research opportunities on alternative factors mediating the relationship between ED, hypogonadism, and MetS.

## Figures and Tables

**Table 1:** Population characteristics.

	All participants (*n* = 151)	Participants according to MetS status		P*
		Participants with MetS (*n* = 74)	Participants without MetS (*n* = 77)	
Age (years), median (P25–P75)	56.0 (50.0–62.0)	56.0 (52.0–62.0)	58.0 (48.0–62.0)	0.790
Weight (kg), median (P25–P75)	80.0 (72.0–86.0)	82.0 (77.0–94.0)	75.0 (68.9–83.5)	<0.001
Height (cm), median (P25–P75)	179.0 (164.0–173.0)	170.0 (163.0–173.0)	169.0 (164.0–173.0)	0.799
BMI (kg/m^2^), median (P25–P75)	27.9 (25.2–30.5)	29.7 (27.7–31.4)	26.3 (24.4–28.3)	<0.001
Waist circumference (cm), median (P25–P75)	104.0 (97.0–111.0)	107.7 (103.0–113.5)	99.5 (95.0–106.0)	<0.001

*Hormonal panel *				
Total testosterone (ng/mL), median (P25–P75)	4.5 (3.5–5.6)	4.0 (3.2–5.0)	5.1 (4.1–5.9)	0.001
Free calculated testosterone (ng/mL), median (P25–P75)	0.102 (0.074–0.156)	0.122 (0.074–0.653)	0.093 (0.075–0.113)	<0.001
SHBG (nmol/L), median (P25–P75)	35.8 (27.3–48.6)	33.6 (23.8–41.6)	40.2 (31.4–55.0)	0.003
LH (mUI/mL), median (P25–P75)	4.0 (2.7–5.9)	4.1 (3.1–7.6)	3.7 (2.6–5.4)	0.062
Estradiol (pg/mL), median (P25–P75)	28.5 (22.0–37.8)	26.0 (21.0–35.4)	31.0 (23.2–40.0)	0.164
FSH (mUI/mL), median (P25–P75)	5.0 (3.7–7.3)	5.1 (3.7–8.1)	4.6 (3.5–6.1)	0.164
TSH (*µ*U/mL), median (P25–P75)	1.3 (1.0–1.8)	1.3 (0.9–1.8)	1.3 (1.0–1.7)	0.634
T3 (pg/mL), median (P25–P75)	3.0 (2.8–3.3)	3.0 (2.8–3.3)	3.0 (2.7–3.3)	0.404
T4 (ng/dL), median (P25–P75)	1.0 (0.9–1.1)	1.1 (1.0–1.2)	1.0 (0.9–1.1)	0.040

*Comorbidities *				
Hypogonadism, *n* (%)	35.0 (23.2)	17 (23.0)	18.0 (23.4)	0.953
Hypothyroidism, *n* (%)	0.0 (0.0)	0.0 (0.0)	0.0 (0.0)	—
Hyperthyroidism, *n* (%)	1.0 (0.6)	0.0 (0.0)	0.0 (0.0)	—
Hyperprolactinemia, *n* (%)	183 (8.2)	—	—	—
Alcohol intake, *n* (%)				
Absent	70.0 (61.9)	25.0 (67.6)	45.0 (59.2)	0.541
Frequent	41.0 (36.3)	12.0 (32.4)	29.0 (38.2)	
Former intake	2.0 (1.8)	—	2.0 (2.6)	
Smoking status, *n* (%)				
Never smoked	55.0 (36.7)	25.0 (33.8)	30.0 (39.5)	0.001
Smoker	32.0 (21.3)	9.0 (12.2)	23.0 (30.3)	
Former intake	63.0 (42.0)	40.0 (54.0)	23 (30.3)	

*Participants with MetS versus participants without MetS.

Data are expressed as the 25th percentile–the 75th percentile (P25–P75).

Metabolic syndrome (MetS), body mass index (BMI), Sex-hormone-binding globulin (SHBG), luteinizing hormone (LH), follicle-stimulating hormone (FSH), thyroid-stimulating hormone (TSH), free triiodothyronine (T3), and free thyroxine (T4).

**Table 2:** Hypothalamic-pituitary-gonadal axis response to metabolic syndrome (MetS).

	Participants with MetS	P*	Participants without MetS	P*
*Without hypogonadism *				
Raised LH levels, *n* (%)				
Yes	6.0 (10.7)	0.903	3.0 (5.2)	0.909
No	50.0 (89.3)		55 (94.8)	

*With hypogonadism *				
Raised LH levels, *n* (%)				
Yes	2.0 (11.8)	0.903	1.0 (5.9)	0.909
No	15.0 (88.2)		16.0 (94.1)	

*Participants with raised LH levels versus participants without raised LH levels.

LH levels were considered normal from 1.7 to 8.6 mUI/mL. Hypothalamic-pituitary-gonadal (HPG) axis was considered to be disrupted when the LH levels were not raised in individuals with hypogonadism.
